# New Horizons in Cholangiopancreatoscopy: Where Are We Heading in 2026?

**DOI:** 10.1111/den.70213

**Published:** 2026-06-22

**Authors:** Raymond Shing‐Yan Tang, Ting Ting Chan

**Affiliations:** ^1^ Department of Medicine and Therapeutics The Chinese University of Hong Kong Hong Kong China

1

After non‐invasive imaging, endoscopic retrograde cholangiopancreatography (ERCP) is the preferred treatment modality over surgery or interventional radiology procedures in many benign or malignant pancreatobiliary diseases [1, 2]. While conventional ERCP‐based techniques for stone extraction, tissue sampling, and stenting of biliary/pancreatic duct strictures are often sufficient in straightforward cases, management of difficult bile duct/pancreatic duct (PD) stones and indeterminate biliary strictures remains challenging in clinical practice [1, 3]. In such situations, advanced diagnostic and therapeutic procedures by peroral cholangioscopy (POCS) or peroral pancreatoscopy (POPS) would be necessary since POCS and POPS allow tissue acquisition and intraductal lithotripsy under direct visualization [4, 5, 6].

In this issue of *Digestive Endoscopy*, Ogura T et al. conducted a comprehensive review of recent advances in cholangiopancreatoscopy [7]. In current clinical practice, cholangiopancreatoscopy can be performed by 1 of the following techniques: (1) two‐operator mother‐baby system, (2) direct cholangiopancreatoscopy with an ultra‐thin gastroscope or a multi‐bending ultra‐slim scope, or (3) digital single‐operator system [8]. While excellent image quality and the capability of image‐enhanced endoscopy (IEE) are the strengths of the mother‐baby system using a dedicated baby video cholangiopancreatoscope, the technically demanding nature of the two‐operator mother‐baby system often precludes wide adoption of this technology and prompts the development of single‐operator cholangiopancreatoscopy (SOC) systems [3]. As technology advances, the relatively easy‐to‐set‐up digital SOC has replaced the legacy fiber optic system and is the most commonly utilized technique of cholangiopancreatoscopy in contemporary clinical practice [1, 2, 3, 7]. Multiple brands of digital SOC systems are now available, with scope outer diameters ranging from 3.1 to 3.9 mm and working channel sizes ranging from 1.2 to 2.0 mm [7]. Aiming to address the commonly perceived drawback of small specimen size obtained by dedicated forceps used in SOC, a 3.9‐mm SOC with a larger 2.0‐mm working channel has become available recently, which allows the use of a larger dedicated biopsy forceps and has the potential to further improve the diagnostic yield of SOC‐guided tissue acquisition [8]. By contrast, the slimmer 3.1‐mm digital SOC can be advantageous in patients needing POPS‐guided lithotripsy of PD stones in a less dilated main PD.

New innovations have further pushed the boundaries of cholangiopancreatoscopy. Recently, a new digital SOC system (Dragonfly, Medtronic, USA) has been recently developed to overcome the limitations of the current digital SOC design, in which a larger catheter outer diameter is necessary to accommodate a larger working channel. When compared with the traditional digital SOC platforms with 2 control dials providing 4‐way tip deflection, the Dragonfly SOC system utilizes a rotational control mechanism that allows 360° shaft rotation and 90‐degree tip deflection with potential for more refined catheter navigation and lesion targeting. With the “think‐outside‐the‐box” scope rotation/tip deflection control design, this new SOC catheter can include a larger 1.7‐mm working channel without increasing the usual 10.5Fr (3.5 mm) outer diameter of traditional digital SOC systems. In turn, a relatively larger 1.5‐mm dedicated biopsy forceps and a larger 4.5 Fr electrohydraulic lithotripsy (EHL) probe can be used in this SOC system with a 10.5Fr outer diameter. A clinical study evaluating the diagnostic and therapeutic performance of this new SOC system is currently underway (ClinicalTrials.gov ID: NCT07120295).

Apart from improvement in tissue acquisition capability in the newer digital SOC systems, efforts have been made to improve visual diagnosis by digital SOC and probe‐based confocal laser endomicroscopy (pCLE) [7, 9]. Artificial intelligence (AI)‐assisted visual diagnosis by SOC holds promise in enhancing endoscopists' ability to differentiate between malignant and benign biliary strictures. Furthermore, while pCLE for evaluation of indeterminate biliary strictures is traditionally performed by an ERCP catheter‐based technique, the recently reported combined technique of performing pCLE under digital SOC guidance has the potential to further enhance the real‐time virtual histological assessment by pCLE since the target lesion can be more reliably identified by direct SOC identification [9].

It is also worth noting that digital SOC applications are not limited to ERCP‐based procedures in modern pancreatobiliary endoscopy. With advances in interventional endoscopic ultrasound (EUS) techniques, transluminal antegrade cholangiopancreatoscopy using digital SOC can now be performed via an EUS‐guided created route (ESCR), opening up new opportunities to tackle anastomotic stricture or intrahepatic duct stones endoscopically in patients with surgically altered anatomy [10].

In summary, the future for cholangiopancreatoscopy is bright. Thanks to the recent innovations in digital SOC design, rapid development of AI and interventional EUS techniques, advanced endoscopists can anticipate further expansion of the clinical applications of cholangiopancreatoscopy in the management of complex pancreatobiliary diseases in patients with standard and surgically altered anatomy in 2026.

## Funding

The authors have nothing to report.

## Conflicts of Interest

The authors declare no conflicts of interest.

## Linked Articles

This article is linked with Ogura et al. (2026). To view this article, visit https://doi.org/10.1111/den.70141.

## Data Availability

Data sharing not applicable to this article as no datasets were generated or analysed during the current study.
